# Nanoplastic Contamination in Freshwater Biofilms Using Gel Permeation Chromatography and Plasmonic Nanogold Sensor Approaches

**DOI:** 10.3390/nano14151288

**Published:** 2024-07-31

**Authors:** Eva Roubeau Dumont, Francois Gagné

**Affiliations:** Aquatic Contaminants Research Division, Environment and Climate Change Canada, Montréal, QC H2Y2E7, Canada; eva.roubeaudumont@ec.gc.ca

**Keywords:** biofilms, freshwater, nanoplastics, pollution sources, oxidative stress

## Abstract

The worldwide contamination of aquatic ecosystems by plastics is raising concern, including their potential impacts on the base of the food chain, which has been poorly documented. This study sought to examine, for the first time, the presence of nanoplastics (NPs) in biofilms from freshwater streams/rivers. They were collected at selected polluted sites, such as the industrial sector for plastic recycling and production, miscellaneous industries, agriculture, municipal wastewaters/effluents and road runoffs. In parallel, the functional properties of sampled biofilms were determined by proteins, lipids, esterase (lipase), viscosity and oxidative stress. The results revealed that biofilms collected at the plastic industries and road runoffs contained the highest NP levels based on size exclusion chromatography, fluorescence detection and a new nanogold sensor visualization method. Examination of the chromatographic elution profiles showed increased abundance and size of NPs in the 10–150 nm size range at the polluted sites. Biofilms from the plastic industry site had elevated levels of aldehydes (oxidative stress) and lipids compared to the other sites. Biofilms collected at the municipal sites had elevated levels of proteins and esterases/lipases, with a decrease in total lipids. Biofilms collected at agriculture sites had the lowest levels of NPs in this campaign, but more samples would be needed to confirm these trends. In conclusion, biofilms represent an important sink for plastics in freshwater environments and display signs of distress upon oxidative stress.

## 1. Introduction

Plastic materials are considered an emerging threat to ecosystems worldwide due to their occurrence and ubiquity. Plastic-based materials are one of the most widely used consumer materials in our economy because of their apparent stability/inertness, lightness and low cost. As they are inadvertently released in the environment, they degrade into plastic debris from 5 mm diameter down to the nanoscale (1–1000 nm), and the number of particles increases exponentially as the size decreases. Microplastics (MPs, 1 µm–5 mm diameter) are found in virtually all environments, including marine and freshwater ecosystems, sediments, soils and the atmosphere [1,2]. Plastic particles provide a unique and ideal colonization structure for microorganisms due to surface increases, sometimes coined the “plastisphere” [3,4]. Biofilms (epiphyton) represent a community of microorganisms (bacteria, fungi, protozoa and algae), spread on solid surfaces, such as plants, rocks, wood and others, including microplastics and nanoplastics (NPs). Biofilms forming on plastics could influence their degradation, adsorption properties and transport/deposition. Indeed, MNP-contaminated biofilms in sludge were shown to disperse and settle much less, thereby remaining in the soluble fraction of effluents and released in surface waters [5]. Moreover, biofilms could detach and transform contaminants attached to the hydrophobic surfaces of plastics and release them to the surrounding environment, similarly to the Trojan-horse effect observed with other nanomaterials. Although plastic pollution has been addressed in most environments, there are relatively few studies dealing with NPs in freshwater environments, especially in biofilms, due to technical limitations. Because biofilms are ubiquitous in aquatic environments as the form at the base of the food chain, they are easily found on solid surfaces. However, their roles to accumulate/retain and degrade NPs have not been investigated yet.

Biofilms are found in most freshwater environments, thriving on solid surfaces in streams, rivers and lakes. The microbial community is diverse and complex, likely to vary depending on environmental conditions, such as nutrients, organic matter composition (e.g., proteinaceous matter stemming from municipal effluents vs. natural organic matter from soil decomposition of organic matter) and climate [6]. They are considered super-organisms, spreading nearly everywhere, even in extreme environments of low nutrients, pollutants, temperature and salinity [7]. These properties (ubiquity and resilience) make them the perfect candidates as bioindicators of plastic pollution across a broad range of aquatic environments. Recent evidence suggests that MPs could disrupt bacteria communications (quorum sensing), as determined by changes in frequency profiles of bioluminescence in *Aliibrio fisheri* [8], which could disrupt the intercellular communications required for the formation and maintenance of biofilms. Biofilms produce a gel-like extracellular matrix, often referred to as extracellular polymeric substances [6]. They are rich in carbohydrates and proteins and form an interface between the microorganisms and the exterior environment. They can favor the formation and transfer of antibiotic resistance phenotypes between bacteria from horizontal gene transfers [9]. The chemical composition of these gel-like substances could also influence the stability and toxicity of NPs. For example, *Pseudomonas aeruginosa* exposed to polystyrene NPs led to a number of toxic effects, from oxidative stress up to the stimulation of the extracellular polymeric substance matrix [10]. Indeed, NPs at 10 and 50 mg/L readily inhibited nitrate reduction, while denitrification and tricarboxylic acid cycle were enhanced at 0.1 mg/l NPs. At higher NP concentrations (10–50 mg/L), polystyrene (PS) NPs upregulated the expression of genes coding for flagellar biosynthesis and biofilm formation as a response to stress. Both MPs and NPs were shown to induce changes in the antioxidative response, oxidative stress and cell membranes in microbial decomposers from biofilms [11]. The understanding of NP contamination from various sources of pollution (industrial, municipal, agricultural) and basic properties and functions of biofilms would provide helpful information on the fate and effects of plastic contamination. A better understanding of plastic pollution in urban, agricultural and rural environments will identify critical sites at risk of plastic pollution. As a corollary, biofilms could constitute ideal bioindicators of environmental contamination problems as they are found virtually on solid surfaces in freshwater ecosystems.

The purpose of this study was, therefore, to examine the presence of NPs in biofilms collected downstream as various sources of pollution in freshwater rivers. The polluted sites were an industrial plastic sector involved in plastic recycling and production, domestic municipal effluents and municipal effluents receiving industrial waste, agriculture area mainly involved in corn production and streams receiving street runoffs and rainfall drainage systems. NP measurement was achieved by size exclusion chromatography with the selective detection of plastics using fluorescence probes and a novel NP visualization and quantitative methodology using modified plasmonic gold nanoparticle sensors. Biofilms were characterized by proteins, lipids and esterase/lipase activity, viscosity and oxidative stress by the measurement of aldehydes. An attempt was made to relate biofilm properties with NPs contaminated at various sites.

## 2. Methods

Biofilms were collected at the Laboratory of Dr Isabelle Lavoie by Lindsey Yvette Mouatcho (Scientific Research Instuture, Sainte-Foy, QC, Canada) on submerged plants such as *Vallisneria americana*, *Potamogeton pusillus* and *Potamogeton perfoliatus*. Submerged vegetation was harvested using a metal rake and transferred to a steel bucket containing 750 mL of lake water. Epiphyton biofilms were mechanically shaken from the plants and transferred into 1 L glass jars after filtration on a 0.5 mm pore size metal sieve to remove plant debris and large invertebrates. Samples were preserved in coolers in the field and stored at 4 °C in the dark until arrival at the laboratory. Biofilms were sampled from three sampling points (10–20 m apart). The biofilms were then lyophilized and stored at −85 °C until analysis.

### 2.1. Detection of NP in Biofilms

Lyophilized biofilms were thawed at room temperature and resuspended in distilled water to make up a final concentration of 200 mg/mL. A volume of 200 µL of biofilm suspension was mixed with 800 µL water and frozen again at −85 °C overnight. For NP assessments, 200 µL of the thawed suspension was mixed with 250 µL of saturated NaCl (5 M) for 5 min followed by the addition of 400 µL of acetonitrile and mixed for 30 min. The suspension was centrifuged at 2500× for 3 min to allow for phase separation and precipitation of insoluble materials. A volume of 10–20 µL was kept aside for the visual assessment of NPs by the nanogold methodology described below. A volume of 200 µL (acetonitrile fraction) was mixed with 50 µL of 0.2% Tween-20 in 100 mM NaCl and injected into a size exclusion chromatography (Sephacryl S-500, 40 × 1 cm) column equilibrated with 0.2% tween-20 and 1 mM KH_2_PO_4_, pH 7.4 serving as the elution buffer [12]. Fractions of 1 mL were collected and analyzed for absorbance at 280 nm, conductivity and NP evaluation using the molecular rotor probe methodology [13]. Calibration of the column was achieved by injecting various concentrations of 20, 50 and 100 nm polystyrene NPs (Polyscience, Niles, IL, USA) and NaCl (conductivity) for total column volume (Vt) determination. The void volume (Vo) was set at 100 nm polystyrene NPs corresponding to 25% of the Vt. The presence of NPs was determined in the fractions by the addition of 50 µL of 10 µM 9-(dicyanovinyl)-julolidine or DCVJ. Fluorescence was measured at 450 nm excitation and 620 nm emission wavelengths [13]. Since the probe was initially developed for polytstyrene, the relative levels of NPs are expressed as PSNP equivalents. The data were expressed as relative fluorescence units (RFU)/mg dry weight. Standard solutions of 50 nm NPs were used for calibration. The levels of NPs in the 150-10 nm size range (corresponding to Ve/Vt 0.33–0.88) were determined by measuring the sum of DCVJ fluorescence in each fraction/total volume (19 mL) and fluorescence equated to ng PSNPs (50 nm) equivalents normalized to mg dry weight.

The semi-quantitative evaluation of plastic nanoparticles was also determined in the acetonitrile fraction (10 µL) by the nanogold sensor [14]. The gold nanoprobe (nAu) was coated with mercaptoundecanoic acid (MUA) from citrate-coated nAu suspensions (Polysciences, USA) of 10–15 nm diameter size range. This methodology was shown to react with polystyrene (PS), polyethylene terephthalate (PET), polyethylene (PE), and polypropylene (PP)/polyamide (PA) types of NPs. Moreover, the levels of PSNPs were validated using the standard addition method in the biofilms, and we found no major interferences in the assay. This method permits easy visualization of the presence of NPs using a free color detection software for smartphones (color detector; R, B and G detection version 3.0.91). A 20 µL volume of the acetonitrile fraction was added to 200 µL of nAu-MUA (50 mg/mL) and color development was initiated with 5 µL of 0.1 M HCl. The appearance of reddish color from the violet-color (blank) background was determined by picture analysis using a color detector software on a common smartphone (5 Mpixel camera). Standard solutions of PSNPs were used for method calibration. The data were expressed as R-channel color units/mg dry weights.

### 2.2. Biofilm Analysis

The biofilm suspensions were allowed to settle at room temperature for 20 min, and aliquots were taken for the analysis of soluble protein, lipid, viscosity and esterase (lipase) activity. The levels of lipids were determined by the Neutral Red dye as previously described [15]. A 20 µL acetonitrile sample was mixed with 200 µL of 10 µM Neutral dye in phosphate-buffered saline (PBS: 140 mM NaCl, 1 mM KH_2_PO_4_ and 1 mM NaHCO_3_, pH 7.4), and fluorescence was measured at 485 nm excitation and 530 nm emission using a microplate reader (Synergy-4, Biotek Instrument, Winooski, VT, USA). Calibration was achieved by Triton X-100, and the data were expressed as mg lipids/mg dry weight. Viscosity was also determined using the molecular rotor probe (9-(dicyanovinyl)-julolidine) but at 450 nm excitation and 520 nm emission wavelengths. Calibration was achieved with standard solutions of glycerol and the data were expressed as RFU/mg dry weight. For soluble proteins, 50 µL of decanted biofilm homogenates was mixed with 20 µL of NaOH 10 mM and mixed at room temperature for 30 min. Then, 10 µL of the sample was tested for proteins using the Coomassie blue staining for proteins at 595 nm absorbance [16]. Calibration was achieved with standard solutions of albumin and the data were expressed as mg proteins/mg dry weight. The levels of aldehydes, a marker for the oxidation of hydroxylated carbons, were determined using 4-aminofluorescein methodology [17]. Briefly, 20 µL of the homogenate fraction was mixed with 200 µL of 10 µM 4-aminofluorescein in PBS and fluorescence was measured at 485 nm excitation and 530 nm emission in a 96-well microplate reader, as described above. The data were expressed as RFU/mg dry weights.

### 2.3. Data Analysis

The biofilms were collected in triplicate (one at each rock) at each of the 10 sites, as depicted in Table 1. They were categorized in the following 6 groups: plastic industries sector (Plast), agriculture (Agri), municipal (Mun), industrial and municipal (Ind/mun), rainfall overflows (rain OVF) and Saint-Charles (reference/baseline) site. The data were expressed as the mean with the standard error and subjected to a non-parametric analysis of variance. Critical difference between sites and the Saint-Charles (ref) site was determined by the Conover–Iman test. Correlation analysis was performed using the Pearson moment procedure. Significance was set at α = 0.05. All tests were performed using the Systat software package (version 13).

## 3. Results

### 3.1. Nanoplastic Levels

**Figure 1:**
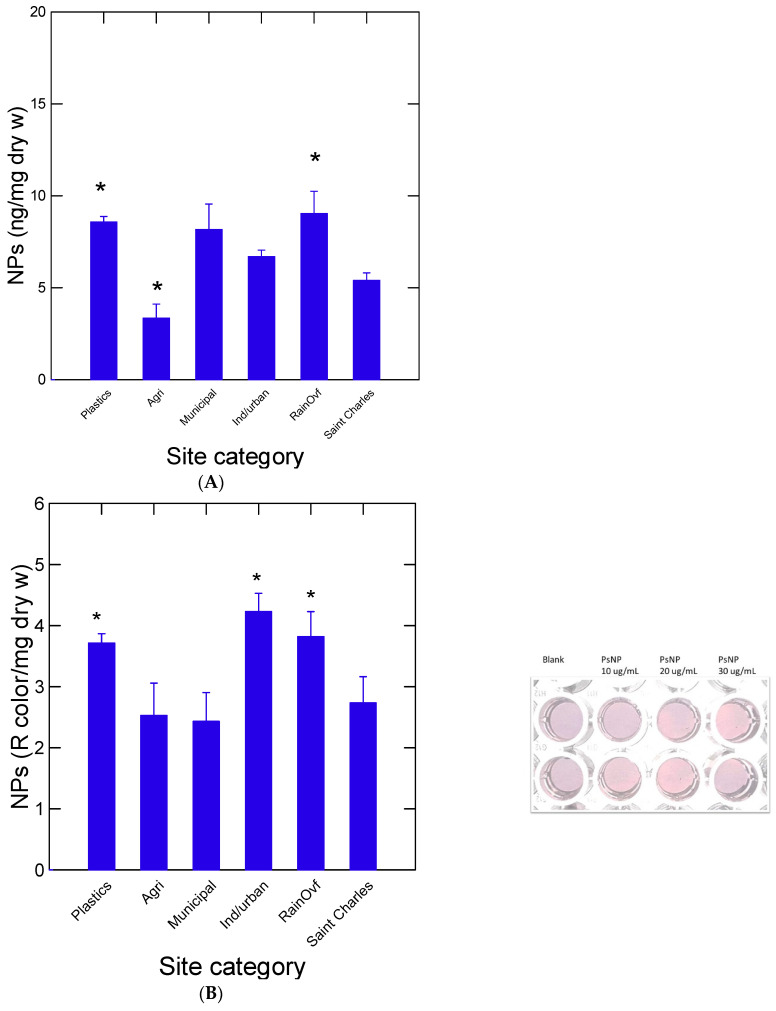
Detection of plastic nanoparticles in biofilms. The measurement of plastic nanoparticles was determined by the coupled size exclusion chromatography and fluorescence by the DCVJ methodology (**A**) and the relative levels of nanoplastics by the nano-gold assay (**B**). The star symbol in (**A**,**B**) represents significance relative to the Saint-Charles site (reference).

**Figure 2:**
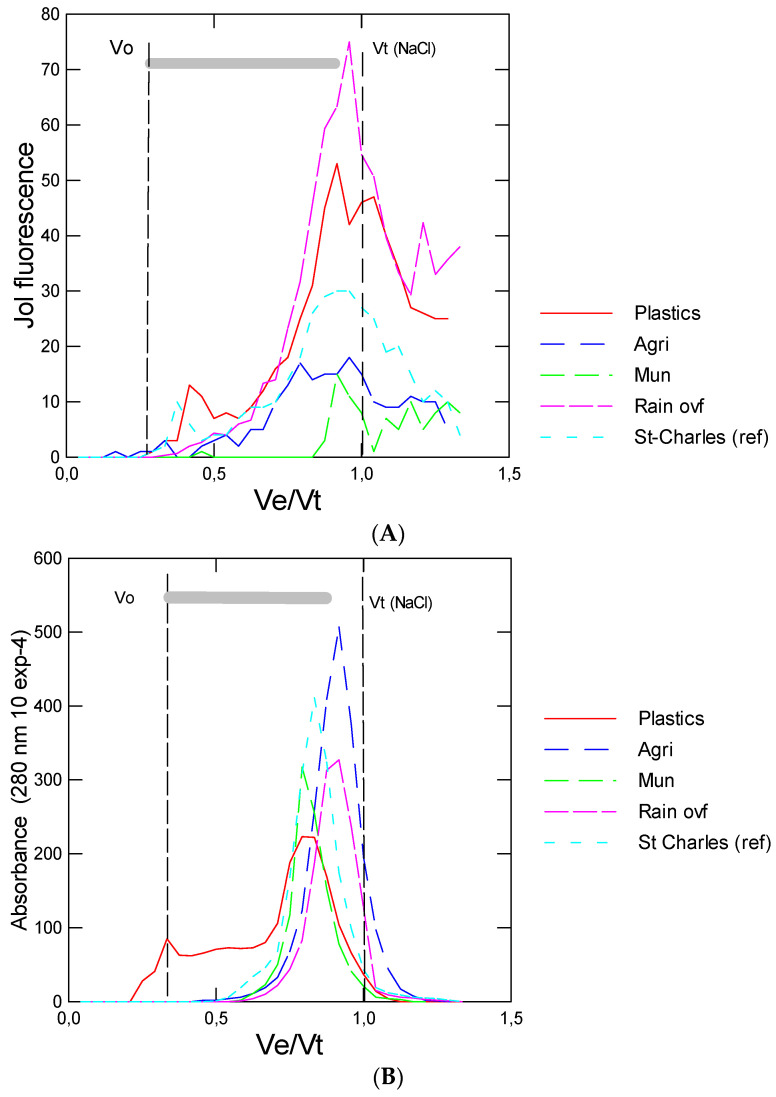
Representative chromatograms of biofilms from various site categories. The DCVJ (Jol) fluorescence e for nanoplastics (**A**) and the absorbance at 280 nm (**B**) were determined. The void volume (Vo) corresponds to the 100 nm PsNP and Vt was determined by NaCl (conductivity). The grey area (bar) corresponds to 100–10 nm size range based on the calibration with the PsNPs.

In this study, 10 sites were utilized for newly formed biofilms on rock substrates (Table 1). The sites were grouped in six categories as follows: plastic (Le Renne river), agriculture (Chibouet river), urban/municipal (Bringham, Brookport in the Yamaska river), industrial/municipal (Grandby township in the Yamaska river), rainfall overflows (Beauport and 2 sites at Cap rouge) and one draining a watershed with few or no industries and large cities (Saint-Charles). This site could be considered a reference or baseline contamination site in the present study. The levels of NPs were determined using size exclusion chromatography and DCVJ staining (Figure 1A). The data showed that NPs were elevated in biofilms formed at the plastic industrial site and rainfall overflow sites. The agricultural site was significantly lower than the Saint-Charles (ref) site. Although the NP levels appeared higher at the municipal and industry + municipal sites, no significant difference was found compared to the baseline site (Saint-Charles). The samples were also analyzed for NPs using a recently developed nAu-MUA methodology for the quick and easy detection of NPs (Figure 1B). The analysis revealed a similar trend; both the plastic industrial and the rainfall overflow sites contained more NPs than the baseline site. However, increased detection was also found for the industrial/municipal site with this assay. Correlation analysis revealed that the nAu-MUA data were significantly correlated with a chromatographic/fluorometric method (r = 0.63; α < 0.01) (Tale 2). This methodology could be used to screen for the semi-qualitative presence of NPs in various samples since the assay requires only a color detector, readily available to smartphone devices. Representative chromatographic profiles of biofilm acetonitrile extracts were provided to evaluate the size distribution of DCVJ fluorescence (NPs) in these samples (Figure 2A). The analysis revealed that the plastic industry and rainfall overflow sites contained high levels of plastic materials compared to the other site categories (agriculture, municipal/industrial and Saint-Charles). Moreover, the size distribution of the elution profiles was larger from the plastic industries and rainfall overflow sites where a peak at the void volume Vo (corresponding to 150 nm) was obtained for the plastic industries site. The largest peak near Ve/Vo = 0.8–0.85 corresponds to smaller particles with diameters in the 10 nm range, which is the size calibration limit of the column. The 20 nm diameter PSNPs were eluted at Ve/Vt = 0.75. Since some plastics absorb at 280 nm (polystyrene, dibutylteraphthalate, petroleum latex, rubber tire nanoparticles, polycarbonates), the elution profiles at this wavelength were also shown (Figure 2B). A broad band encompassing nanoparticles between 150 and 10 nm were detected in biofilms from the plastic industry site. For the other site categories, the absorbance signals were more concentrated at Ve/Vt between 0.85 and 0.9 corresponding to a diameter ≤10 nm. The agriculture and plastic industry sites had larger particles at Ve/Vt = 0.75, corresponding to 20–50 nm sizes based on the calibration with polystyrene nanoparticles (20, 50 and 100 nm). Overall, the DCVJ fluorescent detection of NPs was correlated with absorbance at 280 nm (r = 0.45, α < 0.001), but some sites showed no correlations within: Lerenne, Beauport, Bringham and Yamaska North. This suggests that other non-aromatic plastics (polyethylene, prolypropylene) could also be at play at these sites.

### 3.2. Biofilm Caracterization

**Figure 3:**
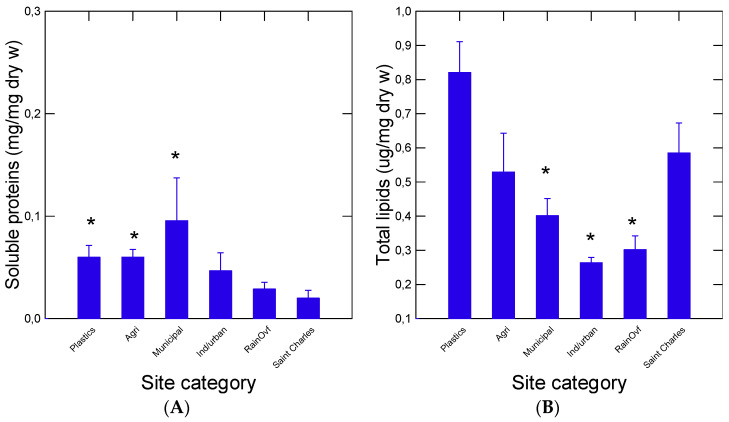
Levels in protein and lipids in biofilms. The levels of soluble proteins (**A**) and lipids (**B**) were determined in biofilm suspension. The data represent the mean with the standard error. The star symbol * represents significance relative to the Saint Charles site.

**Figure 4:**
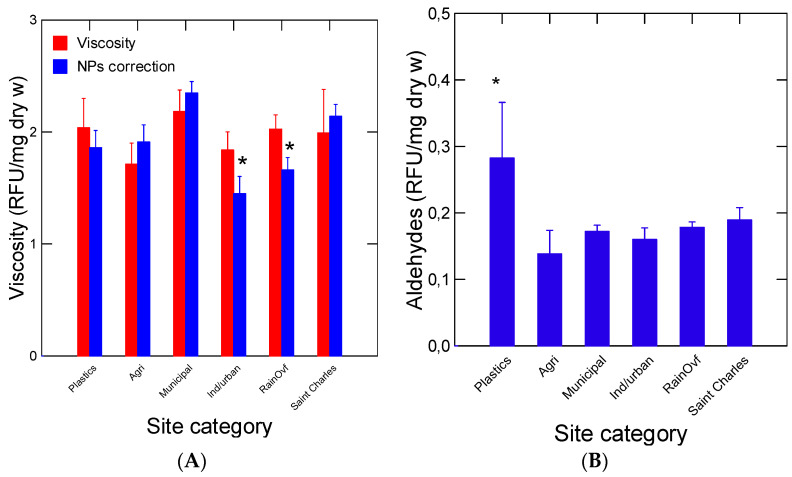

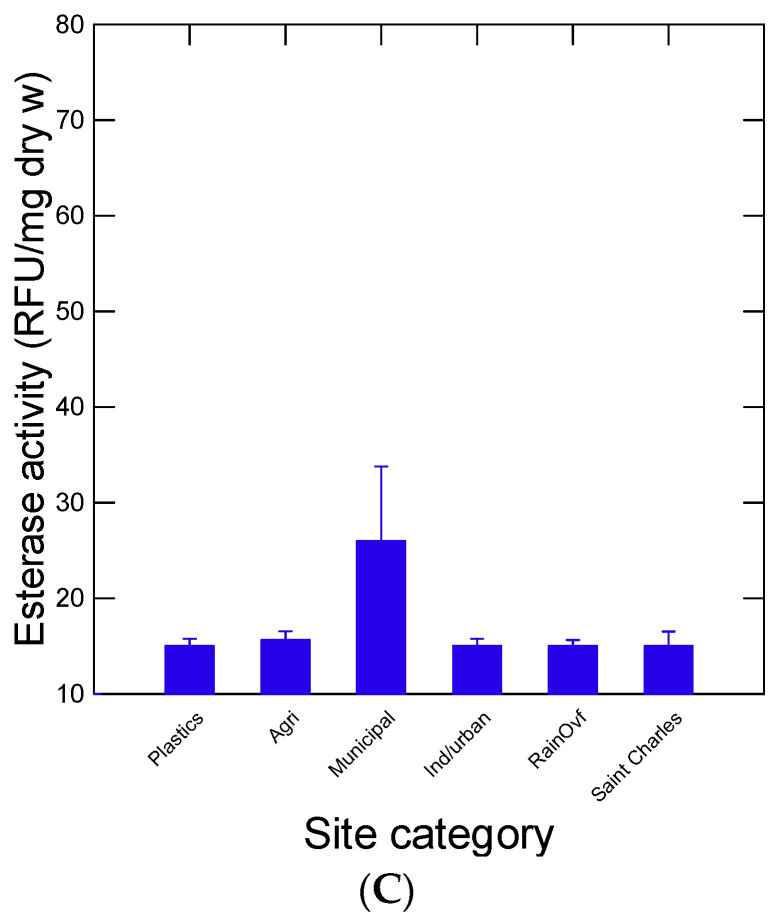
Some biochemical properties of collected biofilms. The viscosity (**A**), levels of aldehydes (**B**) and esterase/lipase activity (**C**) were determined in biofilms. The data represent the mean with standard error. The star symbol * represents significance relative to the Saint Charles site.

In an attempt to characterize the function of biofilms, a number of biomarkers were determined in the decanted water suspensions of biofilms (Figure 3 and Figure 4). Soluble protein concentrations were highest at sites downstream of municipal effluents, agriculture runoffs and the plastic industry sites (Figure 3A). Lipids levels were lower at the industrial, municipal and rainfall runoff sites (Figure 3B). The plastic industry site had high lipid levels compared to most sites but was not significantly different from the baseline site. Soluble proteins and lipid levels were not significantly correlated, suggesting a different origin, perhaps from a different microorganism composition. The biofilm properties were also examined by following the changes in viscosity, esterase (lipase) and for the presence of aldehydes resulting from the oxidation of proteins, plastics and other compounds. No significant changes in biofilm viscosity were observed across the sites (Figure 4A). Total aldehydes were significantly higher at the plastic industry site only (Figure 4B). Esterase activity was significantly higher downstream from the municipal discharge sites (Figure 4C). Correlation analysis revealed that viscosity was significantly related to NP levels detected by both the nAu-MUAC (r = 0.76) and DCVJ (r = 0.7) methods (Table 2). An analysis of covariance of viscosity, corrected against levels of NPs, revealed that both site categories (α = 0.04) and NPs levels (α < 0.001) were significant, and industrial/municipal and rainfall overflow sites were significantly lower than the other sites (Figure 4A). Esterase (lipase) activity was significantly correlated with lipids (r = −0.71, α < 0.01), which were elevated at sites downstream from municipal effluents, suggesting increased lipid hydrolysis in biofilms. A marginal correlation between soluble proteins and esterase activity (r = −0.51; α = 0.1) was also observed (Table 2).

## 4. Discussion

This study sought to determine whether biofilms could be sentinel organisms of plastic contamination in various environments and determine sectors at high risk of plastic exposure. The increased accumulation of plastic-like nanomaterials by biofilms revealed that industries involved in plastic recycling/production and road rain runoffs showed the highest accumulation of NPs in biofilms. Agriculture and municipal effluents exhibited lower plastic contamination based on downstream biofilms draining a rural watershed (Saint-Charles). Given that biofilms are primary decomposers/consumers and widely distributed, one can expect to find interactions between NPs and these microbial communities. It was found that three bacteria strains (*Pseudomonas aeruginosa*, *Bacillus subtilis*, and *Acinetobacter* sp.) forming biofilms were able to sorb up to 716 µg/mg biofilm 430 nm diameter polystyrene NPs [18]. This study also revealed that pH, salinity and temperature had minimal effects on NP sorption in freshwater conditions. In another study, large NPs (>100–1000 nm) and microplastics had negligible effects on chlorophyll a content and functional enzyme activities such as β-glucosidase and leucine aminopeptidase [19]. However, 100 nm diameter polystyrene NPs decreased chlorophyll a and both enzyme activities, suggesting decreased carbon and nitrogen cycling in these biofilms. It was also found that NPs inhibited anaerobic digestion in sewage sludges [5]. In the present study, only phenotypic properties were considered. At a relatively high concentration of NPs (0.2 mg/mL), the relative abundance of *Cloacamonaceae*, *Porphyromonadaceae*, *Anaerolinaceae* and *Gracilibacteraceae* bacteria was lower, while the following families increased due to NPs: *Anaerolinaceae*, *Clostridiaceae*, *Geobacteraceae*, *Dethiosulfovibrionaceae* and *Desulfobulbaceae*. This suggests that biofilm composition is likely to change at various sites impacted by anthropogenic activity including plastic wastes. This is consistent with increased esterase (lipase) activity and soluble proteins with decreased lipids in biofilms found downstream from municipal discharges. However, these changes in properties are not produced by NPs alone since we did not observe strong correlation between the NP concentration and changes in protein, lipid and esterase activity. At the plastic industrial site, increased aldehydes suggest oxidative stress in biofilms. However, NPs were related to the viscosity of biofilms, which could be related to alterations in the extracellular polymeric substances. In biofilms exposed for 14 days to 100 nm NPs, increases in extracellular polymeric substances and filamentous/spirale algae species were observed [20]. Further analysis revealed that NP exposure altered the functional structure, with increased total carbon and nitrogen cycling. Increased nitrogen cycling contributes to the elevation of ammonium in the overlying water. Based on our findings, the production of aldehydes (a marker of oxidative stress) was readily increased in biofilms collected downstream from plastic-related industries. These effects were not observed in biofilms near rainfall/road runoffs. Increased oxidative stress was observed in bacteria exposed to NPs, as evidenced by the increased production of reactive oxygen species [21]. Indeed, *Escherichia coli* and *Bacillus sp* exposed to PsNPs (60 to 220 nm) for 8 h displayed decreased growth and viability where the smallest NPs (60–420 nm) entered cells and enhanced reactive oxygen species production. The production of extracellular polymeric substances was enhanced by the 1 µm diameter particles. The increased production of these extracellular polymeric substances was shown to induce NP aggregation, which could be a protection/detoxification response towards NPs [22]. While increased reactive oxygen species were induced by NPs in diatoms during exponential and stationary phase growth, decreased esterase and other physiological parameters were associated with an increase in polymeric substances (rich in proteins and carbohydrates) during the stationary phase of biofilm growth (*Chaetoceros neogracile*). The production of extracellular polymeric substances in biofilms in response to plastics should be examined more thoroughly. It was shown that polyethylene microplastics increased the production of extracellular polymeric substances in aerobic granular sludge [23]. This led to altered morphology of the aerobic granular sludge, compromising the settling properties and increased biomass release from the reactor to the receiving waters. It was shown that NPs could interact with extracellular polymeric substances via the formation of eco-corona formation at the surface of nanoparticles [24]. These polymeric substances can readily encapsulate plastic and other nanoparticles, thereby modulating surface reactivity, ability to pass in cells and initiated toxicity. Based on the data in this study, the biofilms downstream from the plastic industries site revealed signs of oxidative stress by increased aldehyde levels and NP levels in biofilms. The recent literature suggests that the toxicity of micro-NPs on microbial decomposers involves the antioxidative defense mechanisms, oxidative damage and viability at the membrane permeability level [11]. These effects worsen ecotoxicity due to the presence of existing contaminants adhering to plastics, including plasticizers such as dibutylphthalates and bisphenol A. The presence of antibiotics in municipal effluent could favor the antibiotic resistance phenotype in biofilms attached to plastic materials [9].

It is noteworthy that biofilms collected at rainfall/road runoffs had elevated levels of NPs, as determined by both assays (chromatography/fluorescence detection and nAu-MUA). This could be explained by increased plastic materials resulting from tire wear on nearby roads. The impacts of tire wear nanoparticles on biofilms and other organisms are not well understood at present. Recent studies suggest that biofilms can interact with plastics, producing changes in the community structure and influencing antibiotic resistance gene expression [9]. Biofilms could form directly on plastics and drive the establishment of bacterial resistance genes through horizontal gene transfers. Given that municipal effluents already release most antibiotics destined for humans and livestock, the interaction of these pollutants with plastics, including plastics released from tire wear, in respect to biofilm function needs further examination. Indeed, biofilms collected downstream from municipal sites had elevated levels of esterase (lipase) activity, which was negatively correlated with total lipids in the biofilms. Increased C4 and C8 lipase activity was observed in biofilms forming on mussel shells and in the digestive glands of freshwater mussels exposed to a municipal effluent [25]. This suggests that municipal effluents could alter the bacterial community properties to favor lipid mobilization for energy assimilation. In respect to nanoparticles released from tire wear, our understanding is even lower than plastic waste–biofilm interaction. In a recent study on the characterization of bacterial communities on tire microplastics [3], bacteria colonies readily formed on tire microplastics. The dominant families were proteobacteria (nitrogen fixation) and bacteriodetes (largest group found in gut microbiotia including pathogens), consistent with increased soluble proteins and decreased lipids in biofilm extracts downstream from municipal effluent sites. It was found that nutrients (nitrites, nitrates, ammonium) and dissolved organic matter with temperature and pH were the most important variables driving biofilm formation on tire materials. Synthetic rubbers also release organic chemicals in addition to NPs and contribute to toxicity in marine algae [26]. In this study, the methanol extracts of fifty plastic and elastomer/rubber samples were examined in marine bacteria (bacterial luminescence) and algae. Car tire rubber contained over 2456 chemical features (GC-MS identifiable peaks), while less complex water bottles contained 39 chemical features. Algal and bacterial toxicity was correlated with chemical complexity, and the strongest difference was between thermoplastics (less toxic) and elastomer extracts (tire rubber; more toxic). The toxicity (20% inhibition concentration-IC20) of polyethylene microplastics (1–3 µm diameter) to luminescent bacteria (*Aliivibrio fisheri*) was 2.6 mg/mL after 30 min [8]. However, the examination of luminescence oscillations resulting from communication between bacteria via chemical cues revealed that quorum sensing was altered at concentrations lower than IC20 (≤1 mg/mL) within only a 30 min exposure time. The lack of communication between bacteria could represent one factor contributing to biofilm compactness in granular sludge and the release in effluents/surface waters [5] (Fu et al., 2018). The toxicity of dissolved organic compounds was as toxic to the nanoparticle fraction in *Chlorella vulgaris* (microalgae), *Lemna minor* (plants) and *Daphnia dubia* (microcrustacean) and contributed additively to the toxicity of tire wear leachates [27] (Roubeau-Dumont et al., 2023). Nanoparticles from tire/break wear and fuel combustion were shown to aggregate proteins, leading to amyloidosis, a leading cause of neurodegeneration [28]. Hence, these plastic polymers are found in biofilms and have the potential to disrupt the microbial composition and function in freshwaters. More research will be required to better understand cumulative NPs and existing contaminants in biofilm formation.

In conclusion, this preliminary study highlighted the occurrence of plastic materials in biofilms collected at various sites from urban, agriculture and rural areas. Higher plastic contamination was observed at industrial, urban sites compared to others. High levels of plastic contamination were also observed at sites contaminated by street runoffs/leachates, which suggests the presence of tire and street wear contamination. While lipid levels were somewhat higher at the plastic industrial site, oxidative stress was significantly higher. This study shows that biofilms could represent a sink for plastic contamination in freshwater environments. More research will be needed to better understand the cumulative effects of various plastic sources and the usual contaminants on biofilm functions in aquatic ecosystems. 

## Figures and Tables

**Table 1:** Site location and characteristics.

Sites	Category	Location	Characteristics
Le Renne	Plastics	45.653801, −72.595262	This site is downstream from 3 industries involved in plastics and rubber and 2 from textiles and 1.9 km downstream of a municipal effluent discharge point The treated effluents (aeration ponds) are produced from a town of 5100 inhabitants and wastewaters from a textile industry.
Chibouet	Agri	45.784506, −72.869389	Agricultural area (corn) and receive leachates from cultivation fields.
Yamaska South (Bringham)	Municipal	45.255359, −72.866851	Located 2.1 km downstream the discharge effluents from the city of Brigham (circa 500 inhabitants)
Yamaska North	Industrial/Urban	45.35977, −72.78041	Located downstream of an area supporting 45 industries with 27 ones involved in plastics and rubber and 18 ones for textiles (Granby area). It is located 1.7 km from the discharge point of domestic municipal wastewaters from 39,000 inhabitants.
Yamaska South (Cowansville)	Municipal	45.232789, −72.800473	Located 3.2 km downstream Cowansville city (15,000 inhabitants) and 7 industries involved in plastics.
Beauport	Rainfall overflow	46.865749, −71.209895	Rainfall overflows from the area of Beauport city.
Du Cap Rouge	Rainfall overflow	46.771915, −71.356592	Rainfall overflow site located downstream of a national road (tire wear)
Du Cap Rouge (Tributary)	Rainfall overflow	46.778240, −71.352577	Rainfall overflow collection site
Milette	Rainfall water overflows	46.320774, −72.562209	Rainfall water collector downstream a national road (tire wear).
Saint-Charles	Watershed drainage without direct sources of pollution.	46.910453, −71.371260	Site located from any direct sources of pollution. The river drains an area of 170 km^2^. Considered a reference site in the present study.

**Table 2:** Correlation analysis.

	Lipids	Ald	Viscosity	NP(nAU)	Esterase	Prot	NP(SEC)
Lipids	1						
Ald	**0.57**	1					
Viscostiy	0.04	−0.1	1				
NP(nAu)	−0.11	0.04	**0.54**	1			
Est	−0.07	−0.03	−0.27	−0.37	1		
Prot	0.36	−0.03	0.24	−0.04	−0.02	1	
NP(Sec)	−0.12	0.16	**0.57**	**0.63**	−0.18	−0.08	1

Significant correlations are indicated in **bold**.

## Data Availability

Data are contained within the article.
